# Using Local Norms when Identifying PLD: Utility of Sample Specific Parameters

**DOI:** 10.19070/2332-3000-1600034

**Published:** 2016-10-24

**Authors:** H Lancaster, S Camarata

**Affiliations:** 1 Department of Speech and Hearing Science, Arizona State University, USA.; 2 Department of Hearing and Speech Sciences, Vanderbilt University, USA.

**Keywords:** Elementary, Cultural and Linguistic Diversity, Defining and Diagnosing Disabilities, Speech or Language Impairments, Secondary Data Analysis

## Abstract

This project demonstrates a local norming procedure for ruling out global intellectual delay when identifying primary language disorder (PLD) for children from traditionally underrepresented populations. The Epidemiological Study of Specific Language Impairment Diagnostic Database [9], a population based sample of students with PLD, was utilized for the analysis. Two measures of performance IQ were used to estimate cognitive ability. The database was spilt into Caucasian (n = 1623) and African American (n = 254). Local norms were created using within group z scores. The distributions for the African American group were slightly, but significantly left shifted relative to the normative distribution. After accounting for this left shift during identification, the proportion of African American children in the sample more closely matched the overall population distribution. Creating local norms is a feasible, low-cost solution when dealing with distributions that do not match the normative distribution of a standardized test.

## Introduction

Primary language disorder is a deficit in language comprehension and/or production in the absence of a known etiology, such as hearing loss. Identifying primary language disorder (PLD) requires two components: (a) identification of language deficit and (b) ruling out global intellectual disability [1–5]. In practice, global intellectual disability is ruled out using nonverbal or performance intelligence assessments [5, 6]. Because traditional intelligence tests often have high verbal loads [6], nonverbal, low verbal, or “performance” measures are used to control for the effect of language on the estimate of overall intellectual ability [3]. However, it is not uncommon for a sample’s score distribution to be misaligned with the population normative sample distribution for standardized tests. A well-known example of such a sample distribution shift is the performance of children from minority backgrounds on intellectual tests [7, 8]. Because it is crucial for clinical and educational purposes to accurately identify clinical populations, there is a clear need to provide procedures to address the issue of ruling out global intellectual disabilities when diagnosing children with PLD. Moreover, the problem of how best to rule out global intellectual disability in children from culturally diverse backgrounds remains problematic.

A potential solution to this conundrum is to statistically control for test bias and adjust cut points accordingly, but such an approach requires epidemiologically derived target conditions to estimate the distribution of the skill set in cultural groups. Fortunately, Tomblin and colleagues completed an Epidemiological Study of Specific Language Impairment (EpiSLI) that can illustrate the proposed technique [9]. This population sample of PLD can be utilized to test the effects of statistically generating “local norms” for ruling out global intellectual delay when identifying language impairment. More importantly, if successful, this approach can be applied more widely when relevant data are available. This paper demonstrates a straightforward and low cost solution to ruling out global intellectual delay when identifying PLD in children from minority populations. It is noteworthy that Tomblin, Records and Zhang [10] employed a similar approach to adjust standardized test scores on language measures.

## Local Norms

For adjusting assessment materials to demographic features of local populations, a possible solution is to statistically create local norms with respect to demographic variables. Local norms ensure children are being compared to peers in their community, which would more accurately reflect children’s abilities relative to peers because local norms allow researchers to statistically correct for differences in the local population relative to the normative sample. Local norms also allow the utilization of the procedures proposed by Tomblin, Records and Zhang [10] regarding the use of cut-off scores for marginal means when identifying PLD. In the EpiSLI database, Tomblin, Records and Zhang [10] created local norms specific to his sample for standardized language measures, affecting only 4% of the sample. Specifically, a total of 85 children changed from language disordered to typical language without changing cognitive status when local norms were applied to language parameters. This same logic (and statistical approach) can be applied to any standardized assessment, including the norm referenced intellectual assessment needed to rule out global intellectual disability. Thus, the current study replicates the principles Tomblin et al., used for creating local norms for language assessment in cognitive measures with respect to a demographic variable associated with cultural and linguistic diversity, namely racial/ethnic demographic background.

## Purpose

This study demonstrates a straightforward statistical procedure within a demographically defined group within Tomblin’s sample, African American children, to illustrate this approach in the relative proportion of children identified with PLD or with broader language and intellectual deficits. Because this study applies the approach for language ability by Tomblin, Records and Zhang [10] to cognitive abilities, the same database (EpiSLI) [9] was used. The EpiSLI sample includes sufficient numbers of children with and without PLD from the demographic group of interest (African American) to yield interpretable results. This paper addresses the following research questions:

1. Does the distribution of scores on cognitive measures for African American children differ from that of the normative distribution for the WPPSI?
2. Does the distribution of these scores differ across culturally defined demographic groups within the EpiSLI database?
3. Does statistically adjusting the “cut off ” score for PLD significantly alter or shift the number of African American children in the specific language impaired or low cognitive groups?

The goal of this analysis is to illustrate the practical application of a local norming approach within a well-known sample of children from diverse cultural backgrounds and to extend the findings by Tomblin and colleagues.

## Methods

The purpose of this study was to apply a local norming approach for performance measures of cognitive ability and evaluate the impact of these adaptations to the identification of PLD in the EpiSLI sample of African American children. Specifically, we hypothesized that local norms would classify a higher proportion of African American children as PLD while decreasing the proportion identified as cognitively delayed. The Institutional Review Board at Vanderbilt University approved this study.

## Database

The EpiSLI database is publically available [9, 11]. Tomblin, Records, Buckwalter, Zhang, Smith and O’Brien [11] indicated that the sample matched the 1990 Census data racial make-up, which reported that the population was 80 % Caucasian, 12 % African American, and 7.5 % other minorities. The EpiSLI sample included 84.14 % Caucasian and 13.17% African American, and 2.69 % other minorities, including Hispanic (1.19%), American Indian (0.52%), Asian (0.88%) and unknown (0.10%). Participants were recruited from urban, suburban, and rural school districts in Iowa and Indiana.

Tomblin, Records and Zhang [10] devised a diagnostic standard in this epidemiological study, which was subsequently employed for longitudinal research [12]. In keeping with diagnostic practice, this method utilizes standardized measures of language, performance IQ, and a narrative language task. Tomblin, Records and Zhang [10] diagnostic classifications included four typologies: Typically Developing (TD), Low Normal (LN), Specific Language Impaired here referred to as Primary Language Disorder (PLD), and Nonspecific Language Impaired (NLI). Children were divided on composite language scores into typical language (TD and LN) and language disorder (PLD and NLI) classifications. Tomblin, Records and Zhang [10] required children to have at least two language composite scores below a −1.25 z score to be classified as language disordered (PLD or NLI). Children were also split using performance IQ scores into high (including both TD and PLD) and low (including both LN and NLI) cognitive groups. To be classified as having high cognitive status, children displayed IQ scores of 87 or greater, whereas low cognitive status was identified as displaying scores below 87 but above 70. Thus, children with normal language were classified as TD if they had a performance standard score above 87, or LN if they had a performance IQ less than 87 but greater than 70. Children with language disorders were classified as PLD if they had a performance IQ standard score above 87, or NLI if they had a performance IQ less than 87 but greater than 70. Table 1 provides a visual array of Tomblin’s classifications, which is the system used in this study as well.

The data from the diagnostic battery and demographic data were obtained and linked via identification number from the screening data. Only cases that had completed the diagnostic phase of the EpiSLI study were included in our analysis (n = 1929). Performance IQ measures included the two WPPSI-R subtests and diagnostic status (PLD, NLI, etc.), and demographic classification (e.g., Caucasian, African-American).

### Subjects

Subjects were first classified as TD (n = 1226), LN (n = 198), PLD (n = 277), or NLI (n = 228) based on [10] diagnostic system. There were no differences between the groups for age or gender. Descriptive variables for all four diagnostic groups are presented in Table 2.

A total of 254 African American participants were available in the EpiSLI database and were included in our analyses. Under ideal conditions a parallel analysis on children from other demographic groups (i.e., Hispanic, American Indian, Asian, and unknown) would have been carried out; however, there were insufficient numbers of children in these groups (n = [2, 23], total n = 52). Furthermore, nonparametric analysis of medians indicated no difference for Block Design (p = .212, d = 0.32), but a significant difference for Picture Completion (p = .038, d = 0.38) between Caucasian and other minorities. Therefore these groups were removed from analyses rather than being included as a separate group or collapsed into the Caucasian group.

### Performance IQ

Tomblin, Records, Buckwalter, Zhang, Smith and O’Brien [11] used Block Design and Picture Completion from the WPPSI-R [13] to assess performance intelligence. In Block Design, children are shown a pattern and asked to recreate that pattern using the blocks given to them as quickly as possible. For Picture Completion, children identify what is missing in a picture (e.g., an ear from a cat).

Block Design (r = 0.80) and Picture Completion (r = 0.82) both have acceptable stability [13]; concurrent and predictive validity is not available for subtests. The abbreviated performance intelligence score using only Block Design and Picture Completion has been compared to the full performance measures from the WIPPSI and is a moderately reliable estimate of overall performance intelligence (r = 0.73) [11].

## Analyses

The aim of this study was to apply local norms to a large peer group on classification of language impairment (PLD and NLI) in the EpiSLI database. This study had two phases of analysis. The first phase addressed research questions 1 and 2 by examining the structure of the data. Understanding the data structure was required to identify an appropriate mathematical transformation for creating local norms. The second phase was to create local norms, and then re-classify the target demographic group (African American Students) using Tomblin’s criteria using these norms, and finally to compare the transformed results to untransformed database. The second phase addressed research question 3.

For the first phase, means and standard deviations were compared to the normative information for Caucasian and African American children. The normative mean was a scaled score of 10 and the standard deviation was 3. In quotient units, a scaled score of 10 on a subtest is equivalent to a quotient of 100, whereas a score of 7 would correspond to a quotient of 85. Effect sizes were calculated using Cohen’s d between the group’s mean and the normative mean. Skew and kurtosis were inspected to determine if any distributions were non-normal. Groups were compared to each other using Kruskal-Wallis rank sum test and Cohen’s effect size with pooled variance.

For the second phase we created within group z scores based on race/ethnicity. The within group z scores were used to: (a) calculate the appropriate averages and (b) reassign cases using Tomblin’s guidelines [10] based on local norm cut-offs rather than population norm cut-offs. Therefore, the actual distribution of scores from the local sample of African American children was used to create an autonomous normal distribution and the database subgrouping as TD, LN, PLD and NLI was re-examined using cut-off scores derived from the distribution for African American children.

In accordance with Tomblin’s classification criteria, we defined cognitive status as Typical (greater than or equal to −0.99; e.g. a quotient above 87) or Low Normal (less than −0.99, below 87) using the average z score for Block Design and Picture Completion. Language disordered status was defined as less than or equal to −1.25 z scores on two or more marginal means using the Tomblin, Records and Zhang [10] system. These two criteria were used for all the reclassification examples presented below.

Children were reclassified into one of the four diagnostic groups, TD, LN, PLD, or NLI. Racial proportions were examined in the new samples for each subgroup (TD, NL, PLD and NLI) and statistically compared to the original classifications. The local norming process for intellectual ability allowed children to be reclassified into high and low cognitive groups, but retained their original language status from the EpiSLI database. Thus, a NLI child could be reclassified as PLD, but not LN, and a LN could be reclassified as TD, but not PLD. This process was similar to creating population norms on any standardized test, but using the local sample as the “standard” group.

Chi-square tests were used to determine if there was a difference in proportions within reclassified groups before and after applying the local norms. The original proportions from the EpiSLI database were entered into the Chi-Square as the “expected values,” whereas the proportions after applying the local norms were entered in as the “observed values.” Chi-square tests were used to test for any changes in the proportion of Caucasian and African American in the TD, LN, PLD and NLI groups.

## Results

### Distributions on Performance IQ Subtests Compared to Normal Distribution

**Figure 1.**
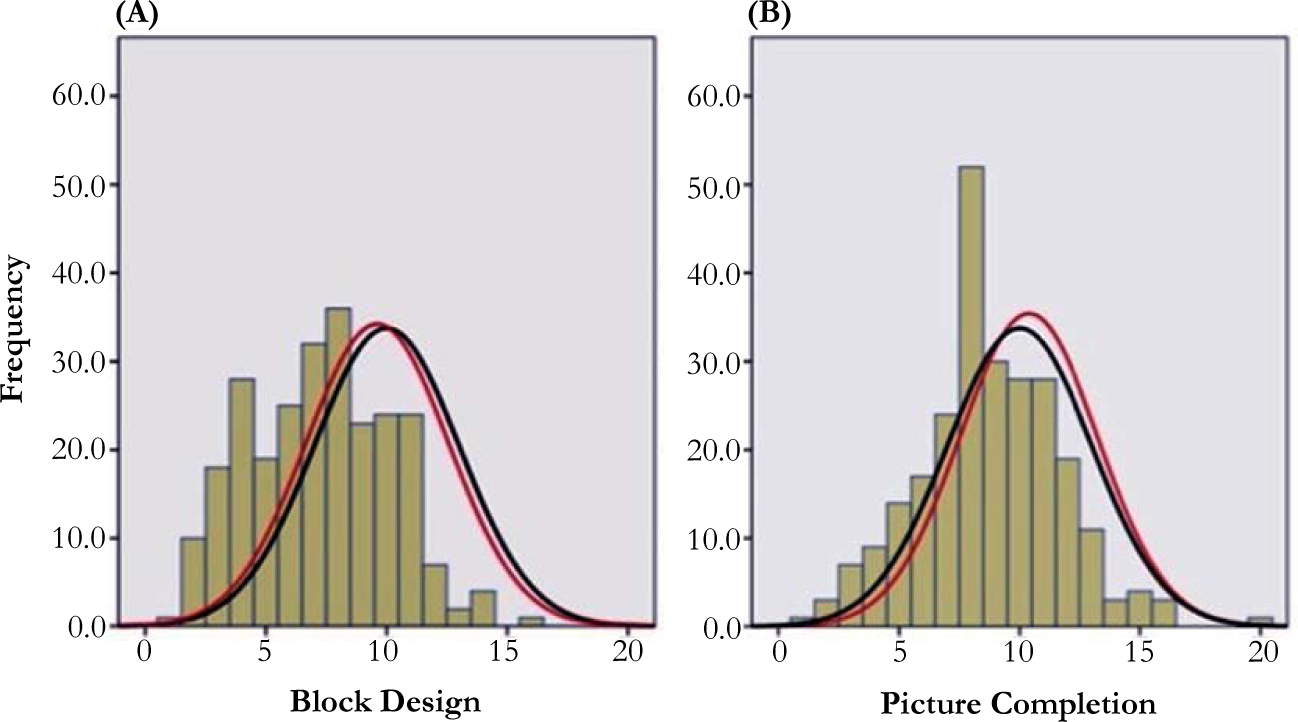
Frequency histogram for African American children (bars) overlaid with Caucasian (red line) and normative (black line) distributions. (A) Block Design and (B) Picture Completion.

African American children differed meaningfully from the normative mean for Block Design (M = 7.23; SD = 2.93; d = 0.93) and Picture Completion (M = 8.74; SD = 2.93; d = 0.43) and the scores from the local sample were normally distributed (Block Design skew = 0.12, kurtosis = −0.55; Picture Completion skew = 0.12, kurtosis = 0.56). Visual inspection of the distribution confirmed normal distribution (Figure 1). In contrast, Caucasian children displayed no meaningful difference from the normative sample for Block Design (M = 9.61; SD = 2.96; d = 0.13) nor Picture Completion (M = 10.38; SD = 2.86; d = −0.13). This is not surprising given that roughly 82% of the normative sample was Caucasian and thus reflected the general test norms.

### Distributions on Performance IQ Subtests Compared Within EpiSLI

The Chi-Square analyses indicated that the African American children significantly differed from the Caucasian children for Block Design (χ^2^ = 121.35; p< .001) and Picture Completion (χ^2^ = 67.46, p< .001). The effect sizes were large (Block Design d = −0.81) and medium (Picture Completion d = −0.57).

### Local Norming

Anchoring within cognitive abilities changed the group status for 160 (8.3%) children from either LN to TD (n = 82) or NLI to PLD (n = 78). Of these 160 children, 85 were African American students, accounting for 33.46% of the total number of African American students. Chi-square tests revealed that PLD group was significantly different from the expected values for African American children after reclassification (23.31%, p< 0.01). The other diagnostic groups did not significantly differ from the full database (LN = 6.96%; NLI = 14.77%; TD = 10.77%). See Table 3 for sample sizes after local norming. Stated directly, a significantly smaller proportion of children from African American backgrounds were identified as “low” cognitive when the local norms procedure was applied to the sample.

## Discussion

Both African American and Caucasian children conformed to Gaussian shaped distributions for the two performance IQ subtest score distributions. However, the African American children’s score distributions significantly deviated from the normal distribution and from their Caucasian peers. These deviations, from the normal distribution and Caucasian peers, highlight the advantage of applying local norms on the African American group. Using local norms decreased the number of African American children classified as low cognitive, regardless of language status. This in turn yielded proportions for diagnostic groups that more closely match the values based on the full EpiSLI database, especially in regards to African American children in low cognitive groups. For the PLD group of African American students, although an increased number of children were shifted from the low cognitive group to the PLD classification, the overall proportion continued to be higher than for the Caucasian group.

Despite the current debate about who should and should not be considered language impaired [14, 15], differentiating PLD from global intellectual delay remains an integral part and key aspect of clinical practice and translational research [1, 4, 5]. However, ruling out global intellectual delay in children from diverse backgrounds can be problematic and, to date, there has not been consensus on how to best address this issue. Although there is a large body of research examining the effect of diverse backgrounds on language assessments [4, 16]; this existing literature has not extended to the issue of ruling out global intellectual disability in these children. This project indicates that the local norms for cognitive measures can be used when identifying language disorders in children from culturally diverse backgrounds and yield distributions more closely matching those seen in Caucasian children. It is particularly noteworthy that applying local norms to the minority group shifted children out of the low cognitive group.

The results of this study demonstrate that applying a statistical approach within the context of local norming yielded reclassification of a portion of African American children within the EpiSLI database, and specifically a decrease in African American children identified as having low overall cognition. We examined the distribution of scores to determine the appropriate method for creating local norms. We created local norms using within group z scores to anchor scores around the groups’ means, thus providing a comparison to a pre-specified peer group. Our results indicate that local norms shifted the proportion of children identified as low cognitive, PLD and TD. The implication of our findings is that local norms impacted the proportion of African American children identified as low cognitive. We suggest that local norms can be created for any demographic variable desired, such as gender, SES, or geographical location.

## Distributions of Performance IQ Subtests

African American children had normally distributed (not skewed), but shifted, distributions on two cognitive subtests of the WPPSI. The distribution of scores for African American children was shifted from the normative distribution by nearly one full standard deviation, but otherwise indicated a Gaussian (bell-shaped) curve. These findings were congruent with previous research for other cognitive measures [17–19].

The effect of these shifted distributions was a higher percentage of African American children in LN and NLI groups compared to the PLD group and the TD group in the unadjusted classification system. This finding parallels what is reported in the schools, wherein higher proportions of African-American children are enrolled in special education, and receive academic support as globally delayed than as language impaired [20, 21] so that applying local norms could, as in this study, shift a significant number of these students into PLD from low cognition.

After utilization of the local norms, the proportions of Caucasian and African American children either stayed congruent (TD) or became congruent (LN and NLI) compared to the overall distribution of the EpiSLI database (i.e., the expected distribution). However, after reclassification, the PLD group was discrepant from the full database after applying the local norms. In other words, African-American children were more likely to be classified as PLD, whereas Caucasian children were more likely to be classified as TD when using local norms. The PLD group started off as not discrepant which may imply that local norms should be used for both language and performance ability. Tomblin’s local language norms affected 85 (4.4%) children, where as we affected 160 (8.3%) by creating local norms for performance ability. Their local norms changed the language status of children, but not the cognitive status. In turn, these local norms changed the cognitive status of the children but not the language status.

## Limitations

The EpiSLI database has been and continues to be a boon to the speech language pathology field, and it must be borne in mind that it was not designed to test the hypotheses proposed for this study. Thus, this study was limited to the participants and variables available in the database: We could apply local norms to the African American students, but not to other minority groups or to other demographic variables such as SES. Previous studies have demonstrated that SES mediates language measures [16] and children from low SES backgrounds score lower by a third to a full standard deviation [22]. We hypothesize that creating local norms using demographic variables tied more closely to SES rather than race will further improve the identification of PLD. It is possible for researchers to obtain both demographic variables and assess the utility of using them together when creating local norms. Future studies should assess the impact of new classification methods when considering race and other demographic factors.

## Conclusions

Tests of performance IQ are used to rule out global intellectual impairment when identifying PLD. These tests measure the same constructs in all children regardless of minority background [23]; however, the score distributions for the African American children in the EpiSLI database were not aligned with the distribution for the normative sample. Furthermore, the distributions between Caucasian and African American children were different for both measures. Because of these two pieces of information, we created distributions specific to peer groups, which changed the number of African American children identified as low cognitive (either LN or NLI). Therefore, local norms are a low-cost solution to correct for violations of distribution assumptions. Local norms only require a large sample size of peers matched for demographic information and computing resources. There are a number of scenarios that local norms would be useful for researchers and clinicians, for example, when testing children with university-educated parents for language impairment.

Clinicians and researchers will most likely continue to consider the impact of global intellectual disability when identifying and treating PLD and continue to use performance measures of cognitive ability in conjunction with language measures. This paper demonstrates how to create and apply local norms: a viable solution for violations of distribution assumptions in circumstances where employing other solutions (e.g., natural language sampling or dynamic assessment) is not feasible because of price or time.

## Figures and Tables

**Table 1.** Representation of Classification System [10].

		Language	
IQ		≤ 1 composites < −1.25 z-scores	≥ 2 composites< −1.25 z-scores
	>85	Typically Developing	Primary Language Disorder
	70 – 85	Low Normal	Nonspecific Language Impaired

**Table 2.** Descriptive Breakdown of EpiSLI Database.

Subsample	N	Age	Gender	Language	Performance	Race
Typically Developing	1226	6 (3.8)	54%	−0.08 (0.67)	0.39 (0.62)	89.3%/8.6%
Low Normal	198	6 (3.7)	53%	−0.55 (0.51)	−1.09 (0.38)	72.2%/22.2%
PLD	277	6 (3.7)	59%	−1.52 (0.38)	0.12 (0.53)	84.8%/12.3%
NLI	228	6 (3.8)	52%	−1.76 (0.48)	−1.27 (0.48)	65.8%/31.1%

Notes. Mean (SD). For Age mean is reported in years and SD in months. Gender is reported as percent male. Language is a composite of five TOLD-P:2 (Newcomer & Hammill, 1988) and two narrative variables (Culatta et al., 1983) as described in Tomblin et al., (1996). Cognition is the average of z-scores of Block Design and Picture Completion (Weschler, 1988). Race is percent Caucasian over percent African American.

**Table 3.** Number of Children Reclassified after Anchoring within Racial Group for Performance IQ.

		Language	
IQ		≤ 1 composites < −1.25 z-scores	>= 2 composites < −1.25 z-scores
	>85	**1309**/*1226*	356/277
	70 – 85	115/198	149/228

Notes. Bolded numbers represent group size after anchoring process. Italics are original values.
